# Homophily and missing links in citation networks

**DOI:** 10.1140/epjds/s13688-016-0068-2

**Published:** 2016-03-03

**Authors:** Valerio Ciotti, Moreno Bonaventura, Vincenzo Nicosia, Pietro Panzarasa, Vito Latora

**Affiliations:** 1 School of Business and Management, Queen Mary University of London, Mile End Road, London, E1 4NS UK; 2 School of Mathematical Sciences, Queen Mary University of London, Mile End Road, London, E1 4NS UK; 3 Dipartimento di Fisica ed Astronomia, Università di Catania, Via S. Sofia, Catania, I-95123 Italy; 4 INFN, Sezione di Catania, Via S. Sofia, Catania, I-95123 Italy

**Keywords:** citation networks, homophily, link prediction, bibliometric techniques

## Abstract

Citation networks have been widely used to study the evolution of science through the lenses of the underlying patterns of knowledge flows among academic papers, authors, research sub-fields, and scientific journals. Here we focus on citation networks to cast light on the salience of homophily, namely the principle that similarity breeds connection, for knowledge transfer between papers. To this end, we assess the degree to which citations tend to occur between papers that are concerned with seemingly related topics or research problems. Drawing on a large data set of articles published in the journals of the American Physical Society between 1893 and 2009, we propose a novel method for measuring the similarity between articles through the statistical validation of the overlap between their bibliographies. Results suggest that the probability of a citation made by one article to another is indeed an increasing function of the similarity between the two articles. Our study also enables us to uncover missing citations between pairs of highly related articles, and may thus help identify barriers to effective knowledge flows. By quantifying the proportion of missing citations, we conduct a comparative assessment of distinct journals and research sub-fields in terms of their ability to facilitate or impede the dissemination of knowledge. Findings indicate that Electromagnetism and Interdisciplinary Physics are the two sub-fields in physics with the smallest percentage of missing citations. Moreover, knowledge transfer seems to be more effectively facilitated by journals of wide visibility, such as Physical Review Letters, than by lower-impact ones. Our study has important implications for authors, editors and reviewers of scientific journals, as well as public preprint repositories, as it provides a procedure for recommending relevant yet missing references and properly integrating bibliographies of papers.

## Introduction

Among the broad category of information networks, including the Word Wide Web [1], email exchange networks [2], and phone call networks [3], the networks of citations between academic papers have been widely investigated to uncover patterns and dynamics of knowledge transfer, sharing, and creation in science [4–7]. The nodes of citation networks are academic papers, each containing a bibliography with references to previously published work. Typically, a directed link is established from one paper to another if the former cites the latter in its bibliography. Because papers can only cite other papers that have already been published, all directed links in citation networks necessarily point backward in time. Citation networks are therefore *directed acyclic graphs*, *i.e.*, they do not contain any closed loops of directed links [8].

Since the seminal work by Derek de Solla Price on the distribution of citations received by scientific articles [6, 7], citation networks have extensively been studied to shed light on the mechanisms underpinning the evolution, diffusion, recombination, and sharing of knowledge over time [9, 10]. The reason why citation networks are crucial to understanding and modelling scientific production is clear. Although citations can serve different functions - for instance, they acknowledge the relevance of previous work, they help the reader of a paper to gather additional information about a specific topic, they point to related work or, sometimes, they can also express disagreement with, or level criticism against, a position endorsed in a paper [11] - the number of citations received is generally regarded as an indication of the relevance and quality of a paper as well as of its authors’ prestige and scientific success [12]. Certainly, citation networks can be used to reconstruct the communication flows among different scientific communities and infer the relation among different research topics and sub-fields [13]. Recent work on citation networks has indeed proposed a new method for highlighting the role of citations as conduits of knowledge. For instance, Clough *et al.* [14, 15] have proposed reduction methods to filter out the relevant citations preserving the causal structure of the underlying network and of knowledge flows.

In this paper, we study citations from a different perspective. First, we assess the extent to which the occurrence of a citation between two papers is driven by the similarity between them. Specifically, we investigate empirically a large data set of articles published in the journals of the American Physical Society (APS) [16], and we measure the similarity between any two articles by drawing on, and extending, a method originally proposed by Tumminello *et al.* in Ref. [17, 18] that enables us to statistically validate the overlap between the bibliographies of the two articles. Results suggest that the number citations made by one article to another is indeed an increasing function of the similarity between the two articles. Our findings thus indicate that the creation of links in citation networks can be seen as governed by *homophily*, namely the principle that similarity breeds connection [19–22].

Second, we propose a novel method for identifying missing links in citation networks. The gist of our argument is simple. We focus on pairs of articles characterised by high degrees of similarity; if a citation between them is missing, we regard the lack of a directed link as a signature of a relevant yet unrecorded flow of knowledge in the network. By uncovering pairs of published articles with missing citations, we rank the APS journals and topics according to the incidence of missing data on knowledge flows.

Our method has important implications for the analysis not only of published articles, but also of newly posted preprints on online archives, or of manuscripts submitted to scientific journals. Specifically, our method can be used to suggest interesting work and relevant literature that could, in principle, be included in the bibliography of recently posted or submitted preprints. As we witness a continuously increasing production of preprints and publication of new articles, it has become particularly difficult for authors to keep abreast of scientific developments and relevant works related to the domain of interest. As a result, lack of knowledge of prior or current related work and missing relevant citations may occur quite often. The method presented in this paper can help the scientific community precisely to address this problem. In particular, it can be used not only by authors to integrate the bibliographies of their work, but also by editors of scientific journals to uncover missing citations and identify the appropriate reviewers for the papers they are considering for publication.

The paper is organised as follows. In Section 2, we introduce and discuss our method for evaluating similarity between articles based on the statistical significance of the overlap between their respective bibliographies. In Section 3, we apply our method to all articles published in the journals of the APS. We show that citations between articles are positively correlated with their similarity, and we then identify missing links between similar articles published in different fields and in different journals. In Section 4, we summarise our findings and discuss implications, limitations, and avenues for future work. Finally, in Section 5, we describe the data set and the validation technique used in our analysis.

## Quantifying similarity between articles

Similarity between two articles can be measured in a number of ways. A straightforward, yet labour-intensive way of comparing articles is to semantically analyse their entire texts. Alternatively, similarity can be simply based on the co-occurrence of a few relevant concepts or keywords in the titles or abstracts of the articles. Moreover, similarity can be measured through the co-occurrence of classification codes, such as those included in the Physics and Astronomy Classification Scheme (PACS), which help identify the research areas to which each article belongs [23]. Here, we propose an alternative measure of similarity based on the comparison between the bibliographic lists of references included in two articles. Our hypothesis is that, if two articles are concerned with related aspects of the same discipline or research problem, then their bibliographies will exhibit a substantial overlap. We shall therefore introduce a method for assessing the statistical significance of the overlap between the lists of references of two articles, and we shall then use the statistically validated overlap as a measure of the similarity between the two articles.

### Overlap between reference lists as a measure of similarity between articles

A natural way to quantify the overlap between two given sets $Q_{i}$ and $Q_{j}$ is the Jaccard index, which is defined as the ratio between the number of common elements in the two sets and the total number of elements in the union of the two sets:

1 $$ J_{ij} = \frac{ \vert Q_{i} \cap Q_{j}\vert }{\vert Q_{i} \cup Q_{j}\vert }. $$

**Figure 1:**
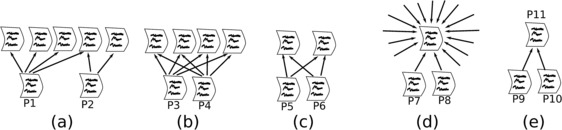
**Quantifying the similarity between two articles based on their bibliographies.** The similarity between two articles can be defined in terms of the overlap between their reference lists. The two articles P1 and P2 in panel **(a)** share only one citation; they should therefore be considered less similar than articles P3 and P4 in panel **(b)** which share four citations. This difference can be captured by the Jaccard index, which is equal to 0.2 in the former case and to 1.0 in the latter. However, the Jaccard index is equal to 1.0 also for the two articles in panel **(c)**, which instead share only two citations. If citations are interpreted as proxies for knowledge flows, then the similarity between articles P7 and P8 in panel **(d)**, which cite a highly-cited article, should be smaller than the similarity between articles P9 and P10 in panel **(e)**, which instead are the only two articles citing P11. Our similarity measure, based on statistical validation, properly takes these heterogeneities into account.

Notice that, in general, if two sets share a higher number of elements, then their Jaccard index will increase, and in particular $J_{ij} = 1$ only if $Q_{i} \equiv Q_{j}$, while $J_{ij} = 0$ if the two sets do not share any element. An example of the suitability of the Jaccard index for measuring the similarity between the bibliographies of two articles is provided in Figure 1(a)-(b). Here the two sets $Q_{i}$ and $Q_{j}$ represent, respectively, the articles in the two reference lists of the two articles *i* and *j*. Since article P1 and article P2 share only one reference over a total of five, their Jaccard index is equal to 0.2. Conversely, the two articles P3 and P4 in panel (b) have a Jaccard index equal to 1.0, since the overlap between their reference lists is complete.

However, the use of the Jaccard index has some drawbacks. First, the value of $J_{ij}$ is always bounded from above by $\frac{\min(\vert Q_{i}\vert , \vert Q_{j}\vert )}{\vert Q_{i}\vert + \vert Q_{j}\vert }$. This means that if the sizes of the two sets are remarkably different, their similarity is primarily determined by the size of the smallest of the two sets. As a consequence, large sets tend to be characterised by relatively small values of similarities with other smaller sets. In addition to this, the Jaccard index does not distinguish between pairs of identical sets having different sizes. In particular, if we consider two identical sets $(Q_{i}, Q_{j})$ of size $N_{1}$ and two other identical sets $(Q_{m}, Q_{n})$ of size $N_{2}$, then we have $J_{ij} = J_{mn} = 1$, regardless of the values of their sizes $N_{1}$ and $N_{2}$. For instance, the Jaccard index of articles P5 and P6 is equal to 1.0 and is identical to that of articles P3 and P4, even though P3 and P4 share a larger number of references. In the case of bibliographic references, this degeneracy of the Jaccard index is very important. In fact, if we interpret references as proxies for knowledge flows from cited to citing articles, then it would be reasonable to associate a higher value of similarity to a pair of articles that share a large number of references than to a pair sharing only few references, since the former pair is expected to draw on a more similar scientific background. In particular, we would expect the two articles in panel (b) to be assigned a value of similarity larger than the two articles in panel (c).

Another drawback of a bare count of the number of common references is that some citations can, in principle, be more important than others. Consider the two cases depicted in Figure 1(d)-(e). In panel (d), articles P7 and P8 have an identical set of references, consisting in the citation to a single highly-cited article. Also in panel (e), both articles P9 and P10 cite the same article. However, in this case the cited article does not receive any citation from other articles. Now, since our aim is to quantify the similarity between articles, a citation to a highly-cited article, such as a review article, should be considered less relevant than a citation to a more specialised or less visible article, which is cited only by articles concerned with a certain specific topic. In other words, it would be preferable to associate a higher relevance to the single citation shared by articles P9 and P10 in Figure 1(e) than to the citation to other highly cited articles shared by articles P7 and P8 in Figure 1(d), and thus to conclude that articles P9 and P10 are more similar than articles P7 and P8.

### Defining statistically significant bibliographic overlaps

The method we propose here allows us to overcome the drawbacks of the Jaccard index discussed above and illustrated in Figure 1. The method is based on an extension of the so-called *Statistically Validated Network (SVN)* approach to the case of directed unipartite graphs. SVNs were introduced by Tumminello *et al.* [17, 18] as a method to filter out statistically irrelevant information from bipartite graphs, such as user-item networks deriving from purchase systems or product reviews. In such systems, a set *A* of nodes (*e.g.*, buyers, users) express preferences over another set *B* of nodes (*e.g.*, books, movies, services). Those preferences or selections are represented by directed links from nodes in set *A* to nodes in set *B*. The idea behind SVNs is that the similarity between two nodes *i* and *j* in the set *A* can be expressed in terms of the co-occurrence of their selections of nodes in *B*, and in particular that it is possible to attach a statistical significance, namely a *p*-value, to each set of common selections made by *i* and *j*.

Citation networks are not bipartite graphs. They are also different from user-item networks because each article in general can only cite other articles that have already been published, and can only receive citations from other articles that will be published after its publication date. Nevertheless, it is possible to draw upon the same idea used to construct bipartite SVNs, and define a similarity between two articles based on the overlap between their reference lists.

Let us consider two sets of nodes, *A* and *B*. The set *A* contains all the articles with more than zero outgoing citations, $A = \{ i \in V | k_{i}^{\mathrm{out}}>0\}$, while the set *B* contains all the articles that have received at least two citations, $B = \{ i \in V | k_{i}^{\mathrm{in}}>1\}$. It is worth noticing that $A\cap B \neq \emptyset$, *i.e.*, the two sets may share some articles, since in general each article cites and is cited by other articles. We denote by $N_{A} = \vert A\vert $ and $N_{B}=\vert B\vert $ the cardinality of the two sets. The method associates a statistical significance to the similarity between a pair of nodes $(i,j)$ in *A* by comparing the number of co-occurrences of citations in their reference lists against the null hypothesis of random co-occurrence of citations to one or more articles in *B*. In this way, the method allows us to identify pairs of nodes in *A* characterised by overlaps between citations to elements in *B* which are statistically different from those expected in the null model.

The method works as follows. For each value *k* of in-degree observed in the citation network, we consider the set of nodes $S^{k} = S^{k}_{B} \cup S^{k}_{A}$, where $S^{k}_{B} \subset B $ contains all $N_{B}^{k} = \vert S^{k}_{B}\vert $ articles with in-degree equal to *k*, and $S^{k}_{A} \subset A$ contains all articles that cite at least one element in $S^{k}_{B}$. Notice that the set $S^{k}$ is, by construction, homogeneous with respect to the in-degree of the elements belonging to the set *B*. Then, for each pair of articles $i,j\in S_{A}^{k}$, we indicate by $d_{i}$ and $d_{j}$ their respective number of citations directed towards the elements of $S^{k}_{B}$. Under the hypothesis that the articles *i* and *j* cite, respectively, $d_{i}$ and $d_{j}$ distinct elements uniformly at random from $S^{k}_{B}$, the probability that they select the same *X* articles is given by the hypergeometric probability function:

2 $$ \mathcal{P}\bigl(X | N_{B}^{k},d_{i} ,d_{j}\bigr) = \frac{{{d_{i}}\choose {X}} {{N_{B}^{k}-d_{i}}\choose {d_{j} - X}}}{{{N_{B}^{k}}\choose {d_{j}}}}. $$

Thus, we can associate a *p*-value to each pair of nodes $i,j\in S_{A}^{k}$:

3 $$ q_{ij}(k) = 1 - \sum_{X=0}^{N_{ij}^{k} -1} \mathcal{P}\bigl(X | N_{B}^{k},d_{i},d_{j} \bigr), $$

where $N_{ij}^{k}$ is the measured number of references that *i* and *j* have in common in the set $S^{k}_{B}$. The *p*-value, $q_{ij}(k)$, is therefore the probability that the number of articles in the set $S^{k}_{B}$ that both *i* and *j* happen to jointly cite by chance is $N_{ij}^{k}$ or more. We repeat the procedure for all possible values of in-degree *k* from $k_{\mathrm{min}}$ to $k_{\mathrm{max}}$, so that each pair of articles $(i,j)$ is, in general, associated with several *p*-values, one for each value of in-degree *k* of the articles in their reference lists. Once all the *p*-values have been computed, we set a significance threshold $p^{*}$ and validate all the pairs of nodes that are associated with a *p*-value smaller than the threshold $p^{*}$. Given a value of the statistical threshold, only the validated pairs of articles are considered similar at that significance level.

However, because each pair of articles $(i,j)$ can be associated with multiple *p*-values, it is necessary to perform hypothesis-testing multiple times. In this case, if we choose a confidence level or significance threshold $p^{*}$, say 1% confidence level ($p^{*}=0.01$), the various *p*-values associated with the same pair of nodes are not compared directly with the chosen significance threshold $p^{*}$, but with a rescaled threshold that appropriately takes the number of tests performed into account. As a method for multiple testing we use the False Discovery Rate (FDR) [17, 24] (see Section 5 for details). Ultimately, we identify the set $\mathcal{M}(p^{*})$ of all pairs of nodes whose similarity is statistically significant at the confidence threshold $p^{*}$. In what follows, we shall denote by $M(p^{*})=\vert \mathcal{M}(p^{*})\vert $ the cardinality of such set. In principle, since each pair of articles $(i,j)$ can belong to different sets $S^{k}$ (and, as a result, can be associated with several *p*-values $q_{ij}(k)$), it would be possible to define a similarity weight $w_{ij}(p^{*})$ for each pair $(i,j)$ as the number of times that the pair is validated at the confidence threshold $p^{*}$. In other words, $w_{ij}(p^{*})$ would be the number of sets $S^{k}$ for which $q_{ij}(k)$ passes the statistical test. However, we do not consider this possibility here, but simply assume that a pair of articles $(i,j)$ belongs to the set $\mathcal{M}(p^{*})$ if at least one of the *p*-values $q_{ij}(k)$ passes the statistical test at the confidence threshold $p^{*}$.

Notice that the definition of the *p*-value associated with a pair of articles in terms of the hypergeometric null model provided in Eq. (2) does not depend on the order in which two articles are assessed. The resulting symmetric value of similarity between any two articles is rooted in the invariance of the hypergeometric distribution in Eq. (2) under permutation of the pair *i* and *j*, *i.e.*, of the two quantities $d_{i}$, $d_{j}$. Moreover, Eq. (2) rectifies some of the problems of measures of similarity based on a bare count of co-occurrences. In particular, two articles that share a small number $N_{ij}^{k}$ of citations will be assigned a higher *p*-value (*i.e.*, a smaller statistical significance of their similarity) than two articles sharing a large number of citations. This means that, for instance, the *p*-value $q_{\mathrm{P3}, \mathrm{P4}}(2)$ associated with the pairs of articles $(\mathrm{P3}, \mathrm{P4})$ in Figure 1(b) will be smaller than the *p*-value $q_{\mathrm{P5},\mathrm{P6}}(2)$ associated with the pair of articles $(\mathrm{P5}, \mathrm{P6})$ in Figure 1(c), since $\mathrm{P3}$ and $\mathrm{P4}$ share a larger number of references (namely, four instead of two) to other articles each receiving two citations. Moreover, the *p*-value associated with the pair $(\mathrm{P7}, \mathrm{P8})$ will be larger (*i.e.*, the similarity between the pair is less statistically significant) than the *p*-value associated with the pair $(\mathrm{P9}, \mathrm{P10})$. The reason lies in the fact that, according to the hypergeometric null model, the co-occurrence of a reference to a highly cited article is more likely to take place by chance than the co-occurrence of a reference to an article with a relatively small number of citations.

## Results

We now show how the proposed method for assigning a statistical significance level to the similarity between any pair of articles based on the statistically validated overlap between the respective bibliographies can indeed turn very useful and help uncover important properties of a citation network.

As an example of the possible applications of the method, we analyse the citation network among articles published in the journals of the APS during the period between 1893 and 2009. The data set is described in detail in Section 5. We shall start by studying empirically the probability $P_{i\to j}(p^{*})$ of the occurrence of a citation from an article *i* to an article *j* validated at a certain statistical threshold $p^{*}$. We shall then discuss how the method can be used to identify missing and potentially relevant references and also to rank journals and scientific topics based on the relative occurrence of missing citations.

### Homophily in citation patterns

We start from the observation that if we consider progressively smaller values of the statistical threshold $p^{*}$, the set $\mathcal{M}(p^{*})$ will shrink and contain only pairs of articles characterised by an overlap between bibliographies that is highly significant, since it has passed a more stringent statistical test. Thus, small values of $p^{*}$ single out pairs of articles that have a highly significant combination of common cited articles. But if two articles share significantly similar bibliographies, then there is a high probability that they are concerned with the same topic or research problem. As a result, it would be reasonable to expect a citation to occur from the more recently published article to the one published at an earlier date. For each value of the statistical threshold $p^{*}$, we computed the number of pairs of articles $M(p^{*})$ validated at that threshold in the APS citation network, and the number $K(p^{*})$ of existing citations between those validated pairs. Then, we define the probability $P_{i\to j}(p^{*})$ that there exists a citation between any two articles whose similarity is validated at the threshold $p^{*}$ as:

4 $$ P_{i\to j}\bigl(p^{*}\bigr) = \frac{K(p^{*})}{M(p^{*})}. $$

**Figure 2:**
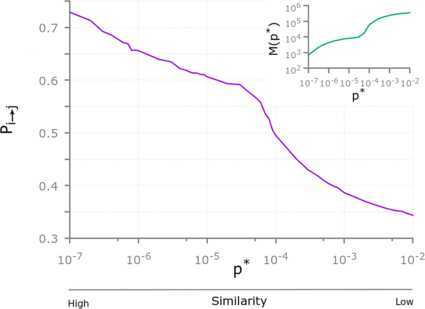
**The probability** $\pmb{P_{i\to j}(p^{*})}$ **to observe a citation between two articles whose bibliographies overlap is statistically significant at the threshold value** $\pmb{p^{*}}$ **.** Notice that $P_{i\to j}(p^{*})$ increases as the statistical threshold $p^{*}$ decreases. That is, citations between pairs of articles characterised by a highly significant overlap tend to occur with a higher likelihood than citations between articles whose reference lists are not significantly similar. The inset shows how the number of pairs of articles characterised by a statistically significant similarity at a given threshold $p^{*}$ varies with $p^{*}$.

The obtained values of $P_{i\to j}(p^{*})$ are reported in Figure 2 as a function of $p^{*}$. The plot clearly suggests that the probability of finding a citation between two articles characterised by a highly statistically significant overlap between the respective reference lists (*i.e.*, the similarity between that pair of articles is validated at a small value of $p^{*}$) is higher than the probability of finding a citation between articles whose reference lists are only moderately significantly similar. For instance, a citation between a pair of articles $(i,j)$ whose overlap between reference lists is validated at $p^{*}=10^{-2}$ occurs only with probability $P_{i\to j}\simeq0.35$, while citations occur between up to 73% of the pairs of articles validated at $p^{*}=10^{-7}$. In other words, the probability that an article *i* cites another article *j* is an increasing function of the similarity between the two articles.

In the social sciences, the principle that similarity breeds connection is traditionally referred to as homophily. This principle has been documented in a variety of empirical domains [19–22]. It is interesting to observe that homophily can also be found to govern citation networks where it plays an important role in shaping the structure and evolution of knowledge transfer between academic papers.

### Suggesting missing references

The identification of a statistically significant similarity between two articles can be used to uncover potentially missing references. For instance, the implementation of a recommendation procedure based on statistically significant overlaps between bibliographies might be useful to assist the editor of a scientific journal in suggesting a list of possibly relevant (and missing) references to the authors of a submitted paper.

**Figure 3:**
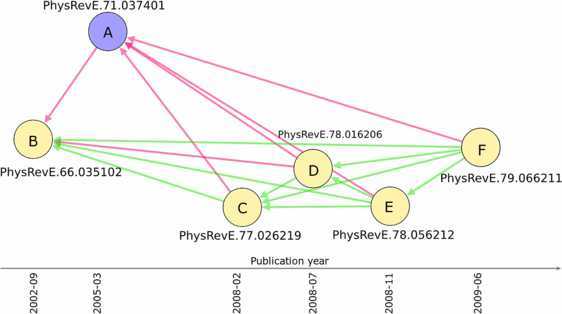
**Lack of knowledge flows.** An example of several validated pairs of articles in the APS citation network at $p^{*}=10^{-7}$. Articles are reported in order of publication time, from older (left) to more recent (right) ones. The occurrence of a link indicates that the pair of articles has passed the statistical test, while the colour of the link indicates that the most recent article in the pair actually did (green) or did not (red) cite the other one. In this case, all the articles represented as yellow nodes are articles co-authored by researchers in the same group, while article A was co-authored by another group. The identification of a large number of missing citations suggests that the two groups might have been unaware of the work of their colleagues in the same field.

Figure 3 shows a typical problem that could be fruitfully addressed through an appropriate reference recommendation system based on the identification of statistically significant overlaps between bibliographies of papers. We report a subgraph of the APS citation network consisting of several pairs of articles validated at $p^{*}=10^{-7}$. Each article is represented as a node, and validated pairs of nodes are connected through a link. The color of each link indicates whether the older article was (green) or was not (red) cited by the more recent one. Note that there is a prevalence of green links, which is consistent with the fact that, for a significance level $p^{*}=10^{-7}$, a citation between a validated pair of articles occurs in more than 73% of the cases (see Figure 2). However, we notice that article A has a considerable number of missing citations, resulting from the fact that it was not cited by any of the four articles that were published after its publication date and with which it shares a statistically significant portion of its bibliography (namely, nodes C, D, E, F). This could mean that either the authors of articles C-F were not aware of the existence of article A, despite the substantial overlap between their reference lists, or that article A was not particularly relevant to the topics addressed in the other articles.

Surprisingly, a more in-depth analysis of the articles in Figure 3 suggests that, not only did all of them appear in the same journal (Physical Review E), but indeed they are all concerned with the same topic (electric discharges) and share a relatively large fraction of PACS codes (05.45.-a, 52.80.Hc). The high degree of similarity between topics can also be easily inferred from the abstracts and introductions of these articles. Interestingly, we found that articles B-F (yellow nodes) were all co-authored by the same research group $G_{1}$, while article A (the only blue node) was the result of the work of a different research group $G_{2}$. The fact that also article A does not cite article B suggests that the researchers in group $G_{1}$ were likely to be unaware of the work conducted by group $G_{2}$ in the same research field, and vice versa.

In this particular case, the quantification of statistically significant overlaps between bibliographies could have been used to facilitate the flow of knowledge between different research groups. For instance, the editor of Physical Review E or the selected reviewers could have brought article B to the attention of the authors of article A, and similarly, when articles C-F were submitted to the same journal, the editor or the reviewers could have advised the authors of group $G_{2}$ to include article A in the bibliographies of their submitted papers.

### Ranking journals and disciplines by (lack of) knowledge flows

So far our analysis has been focused on the whole APS citation network. Physics is a very broad disciplinary area, including sub-fields as diverse as atomic physics, astronomy, particle physics, statistical mechanics, just to mention a few [13]. It is therefore reasonable to perform our analysis of the probability $P_{i\to j}(p^{*})$ at the level of sub-fields. Specifically, we argue that the percentage $P_{i\to j}(p^{*})$ of citations occurring between pairs of articles associated with a similarity that is validated at the statistical threshold $p^{*}$ can serve as a proxy for the knowledge flows taking place within a sub-field. In what follows we restrict our analysis to the six citation sub-graphs induced by the articles that appeared in each of the six research journals published by APS (in order to quantify the ability of each journal to facilitate or impede the dissemination of knowledge), and to the ten sub-graphs associated with the highest levels in the PACS taxonomy (which could shed light on the typical patterns of knowledge dissemination in different sub-fields). The lack of knowledge flows within a journal or a sub-field at a certain confidence level $p^{*}$ can be quantified by the fraction of missing links:

5 $$ U\bigl(p^{*}\bigr) = 1 - \frac{K(p^{*})}{M(p^{*})} = 1 - P_{i\to j}\bigl(p^{*}\bigr). $$

**Figure 4:**
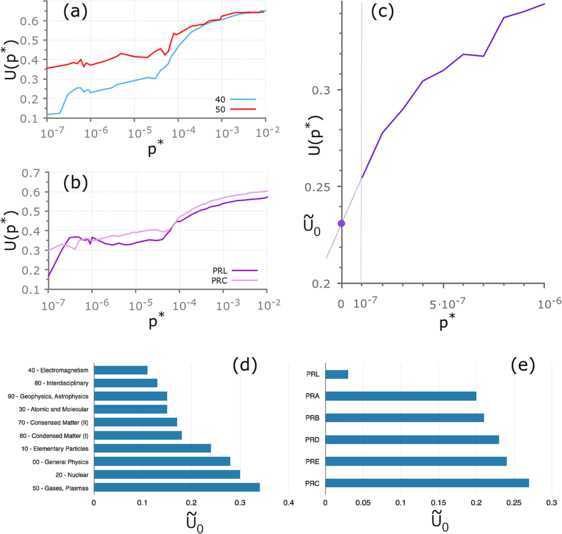
**Ranking journals and sub-fields by lack of knowledge flows.** The analysis of missing links restricted to specific sub-fields of physics or single APS journals confirms that the tendency of a citation to occur between a pair of articles increases with the similarity between the bibliographies of the two articles. Panels **(a)**-**(b)** show the plots of $U(p^{*}) = 1 - P_{i\to j}(p^{*})$ for different sub-graphs corresponding to **(a)** two families of PACS codes, namely 40 (electromagnetism) and 50 (Gases and Plasmas), and **(b)** two APS journals, namely Physical Review Letters and Physical Review C. In panel **(c)** we sketch the procedure adopted to compute the estimate $\widetilde{U}_{0}$: we consider the line tangent to the curve $U(p^{*})$ at the smallest value of the statistical threshold $p^{*}$ for which we still have a relatively substantial number of validated pairs (in this case, $p^{*}=10^{-7}$), and we define $\widetilde{U}_{0}$ as the value of the intercept at $p^{*}=0$ of that line. In panels **(d)** and **(e)** we show, respectively, the rankings of sub-fields and APS journals based on the values of $\widetilde{U}_{0}$. Notice that Electromagnetism and Interdisciplinary physics are the two sub-fields with the smallest percentage of missing links, *i.e.*, those in which knowledge among articles flows effectively and as would be expected if citations were driven by overlaps between topics or research problems. Interestingly, the lack of knowledge flows between articles published in Physical Review C ($\widetilde{U}_{0}\simeq 0.27$) is almost nine times as large as the one identified in Physical Review Letters ($\widetilde{U}_{0}\simeq0.03$), which is the APS journal with the widest visibility and largest impact.

In general, the lower the value of $U(p^{*})$, the more likely it is that a citation occurs between a pair of articles characterised by a similarity validated at the statistical threshold $p^{*}$. Figure 4(a)-(b) shows how $U(p^{*})$ behaves as a function of $p^{*}$, respectively, for all articles whose main PACS code is either in group 40 (Electromagnetism) or in group 50 (Gases and Plasmas), and for all the articles published in Physical Review Letters and in Physical Review C. The figure clearly shows that, even though in all cases $U(p^{*})$ decreases when $p^{*}\to0$, different journals and different sub-fields tend to be characterised by slightly different profiles of $U(p^{*})$, namely by different propensities to obstruct knowledge flows between similar academic papers. A comparative assessment of journals and sub-fields according to their typical ability to facilitate the dissemination of knowledge would, of course, be based on $\frac {K(p^{*})}{M(p^{*})}$. Moreover, the ranking will in general depend on the chosen value of the statistical threshold $p^{*}$.

From a theoretical point of view, a suitable approach to the ranking would be to compute the quantity:

6 $$ U_{0} = \lim_{p^{*}\to0} U\bigl(p^{*}\bigr), $$

namely the limiting value of $U(p^{*})$ when we let the statistical threshold $p^{*}$ go to zero. However, this quantity cannot be computed accurately for a finite network, since for a certain value $p^{*}>0$ the number $M(p^{*})$ of validated pairs at $p^{*}$ will be equal to 0, and the ratio $\frac{K(p^{*})}{M(p^{*})}$ would therefore be undetermined. Here we employ a simple workaround, namely we consider the tangent at the curve $U(p^{*})$ at the smallest value of $p^{*}$ for which the number of validated pairs is still large enough for the construction of a network of a reasonable size (we found that 10^−7^ is an appropriate choice in our case), and we compute the intercept at which this tangent crosses the vertical axis. The value obtained is denoted as $\widetilde{U}_{0}$, and is used as an approximation of $U_{0}$. The procedure used to determine $\widetilde{U}_{0}$ is sketched in Figure 4(c).

In Figure 4(d)-(e) we report the ranking induced by $\widetilde{U}_{0}$ respectively for the ten high-level families of PACS codes (panel (d)) and for the journals published by APS (panel (e)). It is worth noticing that Electromagnetism and Interdisciplinary Physics are the two sub-fields with the smallest percentage of missing links, *i.e.*, those in which knowledge flows effectively among articles (and authors), as would be expected if the occurrence of citations were driven by overlaps between topics or research problems. Interestingly, the rate of occurrence of missing citations in Physical Review C ($\widetilde{U}_{0}\simeq0.27$) is almost nine times as large as the one observed in Physical Review Letters ($\widetilde{U}_{0}\simeq0.03$), which is the APS journal with the widest visibility and largest impact.

## Conclusions

In our study we have proposed a novel method for quantifying the similarity between articles based on their bibliographies. The identification of a statistically significant similarity between articles can be used to uncover potentially interesting or relevant references that are missing from their bibliographies. Our method can thus assist the authors of scientific papers in compiling a list of relevant references, or the editors and reviewers of scientific journals in suggesting otherwise neglected references to the authors of manuscripts submitted for publication. Moreover, public preprint repositories, such as arXiv.org, could automatically quantify the similarity between the bibliography of a newly posted paper and the bibliographies of all other papers in their data set, and then propose a list of papers that the authors might find relevant to their work. The implementation of a recommendation procedure based on statistically significant overlaps between bibliographies might also facilitate the dissemination of scientific results within a scientific field. Problems such as the one shown in Figure 3 can be aptly overcome through the use of our method that enables missing and relevant references to be promptly identified.

Notice that, in our approach, when similarity is evaluated between any two articles published in two different years, all the articles published in the time interval between these two years can only be cited by the more recent article. In principle, it would be possible to modify our method in such a way that the evaluation of similarity would be based only on articles published before the earlier one. However, in this paper, we opted not to take the difference in publication years into account in our similarity measure, because this enables pairs of articles published in different years to be more dissimilar than articles published at the same time, all else being equal. This would result from different opportunities, research directions and resources provided by the different time frames in which the two articles were published. Our method does indeed capture this time-induced dissimilarity between articles. Moreover, since the analysis was based on the APS data set, the evaluation of the similarity between any two articles was restricted to the overlap between the citations the two articles made only to other articles published in the APS journals. The assessment of similarity could not therefore reflect the entire bibliographies of the two articles. This limitation can be easily overcome through further analysis of other citation networks extracted from different data sets, such as ISI Web Of Science, or arXiv.org. Finally, our framework can be extended beyond the domain of citations between academic papers, and be used for uncovering missing and potentially relevant links in other citation networks, such as those between patents [25, 26] or between the US Supreme Court verdicts [14, 27, 28].

## Materials and methods

### The APS data set

Our data set includes bibliographic information on all the articles published by the APS between 1893 and 2009 [16]. The citation graph $G=(V,E)$ includes $|V| = 450\mbox{,}084$ articles, and $|E| = 4\mbox{,}710\mbox{,}547$ directed links. The citations refer only to articles that have been published in APS journals. For each article we extracted the publication date, the main research subject (according to the PACS taxonomy), and its bibliography. Each article belongs to a specific journal. We restrict the analysis to the seven major journals, namely Physics Review A, B, C, D, E and Letter, which are specialised in different sub-fields of physics.

We performed our analysis at three levels, namely the entire citation network, the sub-graphs of the citation network induced by articles in each of the ten main sub-fields of physics, as identified by the highest levels of the PACS hierarchy, and the six sub-graphs induced by articles published in Physical Review Letters and in Physical Review A-E. In our analysis, we discarded articles that appeared in Review of Modern Physics, which publishes almost exclusively review articles. In Table 1 we report the description of the ten main categories in the PACS taxonomy and the topics covered by each of the six journals here considered.

**Table 1:** **The scientific domains associated with the PACS codes and journals**

	**Domain**
PACS code	
00	General
10	The Physics of Elementary Particles and Fields
20	Nuclear Physics
30	Atomic and Molecular Physics
40	Electromagnetism, Optics, Acoustics, Heat Transfer, Classical Mechanics, and Fluid Dynamics
50	Physics of Gases, Plasmas, and Electric Discharges
60	Condensed Matter: Structural, Mechanical and Thermal Properties
70	Condensed Matter: Electronic Structure, Electrical, Magnetic, and Optical Properties
80	Interdisciplinary Physics and Related Areas of Science and Technology
90	Geophysics, Astronomy, and Astrophysics
Journal	
Physics Review A	Atomic, molecular, and optical physics
Physics Review B	Condensed matter and materials physics
Physics Review C	Nuclear physics
Physics Review D	Particles, fields, gravitation, and cosmology
Physics Review E	Statistical, non-linear, and soft matter physics
Physics Review Letter	Moving physics forward

### False discovery rate (FDR) statistical test

The validation of a given pair $(i,j)$ in the FDR method is performed as follows [24]. We set a statistical threshold $p^{*}$ and we assume that there are in total $N_{t}$ tests. Then, the *p*-values of different tests are first arranged in increasing order ($q_{1} < q_{2} <\cdots< q_{N_{t}}$), and the rescaled threshold is obtained by finding the largest $t_{\mathrm{max}}$ such that

7 $$ q_{t_{\mathrm{max}}} < \frac{p^{*} t_{\mathrm{max}}}{N_{t}}, $$

where $N_{t}$ is the number of tests. In this specific case, $N_{t}$ is the number of distinct pairs of articles that are tested over all the sets $S^{k}$ of in-degree classes in the citation network. Then we compare each *p*-value $q_{ij}(k)$ with the rescaled threshold, and we validate the pair $(i,j)$ if $q_{ij}(k) < p^{*} t_{\mathrm{max}} /N_{t}$.
